# Naturally absorbed lithium may prevent suicide attempts and deliberate self-harm while eicosapentaenoic acid may prevent deliberate self-harm and arachidonic acid may be a risk factor for deliberate self-harm: The updated different findings in new analyses

**DOI:** 10.3389/fpsyt.2022.1083739

**Published:** 2022-12-16

**Authors:** Toshihiko Izumi, Masayuki Kanehisa, Takeshi Terao, Ippei Shiotsuki, Masanao Shirahama, Moriaki Satoh, Masaaki Muronaga, Kentaro Kohno, Hirofumi Hirakawa, Masaki Etoh, Takehisa Matsukawa

**Affiliations:** ^1^Department of Neuropsychiatry, Oita University Faculty of Medicine, Oita, Japan; ^2^Psychiatric Medical Center, Oita Prefectural Hospital, Oita, Japan; ^3^Department of Epidemiology and Environmental Health, Juntendo University School of Medicine, Tokyo, Japan; ^4^Department of Forensic Medicine, Juntendo University School of Medicine, Tokyo, Japan

**Keywords:** lithium, eicosapentaenoic acid, docosahexaenoic acid, arachidonic acid, deliberate self-harm, suicide attempt

## Abstract

**Background:**

Since our previous investigation on the effects of trace lithium, eicosapentaenoic acid (EPA), docosahexaenoic acid (DHA), and arachidonic acid (AA) on deliberate self-harm and suicide attempts in 2018, to our knowledge, no replication study has been conducted on this topic.

**Subjects and methods:**

We increased 37 new patients and totally 234 patients were re-analyzed to further investigate the association of suicide-related behaviors with levels of trace lithium, EPA, DHA, and AA in a different way to avoid multicollinearity.

**Results:**

Higher lithium levels were significantly associated with fewer suicide attempts and deliberate self-harm, higher EPA levels were significantly associated with fewer deliberate self-harm, and higher AA levels were significantly associated with more deliberate self-harm.

**Discussion:**

Although the sample size was only slightly larger than the previous study, the present results were clearly different from the previous ones due to the use of different statistical analyses to avoid multicollinearity.

**Conclusion:**

The present findings suggest that naturally absorbed lithium may protect against suicide and deliberate self-harm, while naturally absorbed EPA may protect against deliberate self-harm. However, naturally absorbed AA may be a risk factor for deliberate self-harm.

## 1 Introduction

While omega-3 fatty acids such as eicosapentaenoic acid (EPA) and docosahexaenoic acid (DHA) have been reported to be protective against depression (1–5), EPA rather than DHA has been reported to be effective against depression (2, 3, 6). Recently, however, a large randomized clinical trial investigating 18,353 adults aged 50 years or older without clinically relevant depressive symptoms at baseline revealed that in comparison with placebo, marine omega-3 fatty acid supplementation (1 g/d of fish oil, including 465 mg of EPA acid and 375 mg of DHA) caused a small but statistically significant increase in the risk of depression but no difference in the mood scores over a median follow-up period of 5.3 years, yielding no supportive evidence for the use of omega-3 supplements in adults to prevent depression (7).

Regarding suicide, the effects of omega-3 fatty acids on suicide appear to be inconclusive (8) because large epidemiological studies (9, 10) have shown no supportive evidence for the association between a high intake of fish, EPA, DHA, and lower suicide risk. In addition, the level of arachidonic acid (AA), an omega-6 fatty acid, was reported to be higher among women with a higher risk of suicide (11), whereas bipolar patients who attempted suicide showed lower levels of AA than those who did not attempt suicide (12). Recently, higher AA levels were reported to be associated with greater depression severity at baseline, whereas low AA levels unexpectedly predicted subsequent suicide attempts (13). Although these are conflicting findings, a recent report (14) showed inverse U-shaped (bell shape) curves of the association between depression severity scores and plasma AA, and between brain serotonin transporter binding and plasma AA, which may explain these contradictory findings.

On the other hand, therapeutic doses and levels of lithium have been reported to be protective against suicide in mood disorders (15–18). Although lithium uptake from environmental ingestion (e.g., trace levels in tap water) as measured in serum lithium levels (median 0.00072 mEq/L) (19) is much lower than the recommended level for therapeutic use (approximately 0.4 to 1.0 mEq/L), trace lithium uptake has been reported to be associated with a low suicide rate in epidemiological studies (20–27). However, lithium levels in tap water have been reported to show a negative association with depressive symptoms and interpersonal violence but not with suicidal behaviors among a general population of adolescents (28).

Since previous studies investigated the effects of omega-3, omega-6, and lithium on suicide-related behaviors separately, we comprehensively investigated the effects of EPA, DHA, AA, and trace lithium on deliberate self-harm and suicide attempts with adjustment for each other (29), and our findings showed that higher EPA and log-transformed lithium levels were associated with lower suicide attempts, whereas higher AA levels were associated with higher deliberate self-harm and higher DHA levels were associated with higher suicide attempts. These data were obtained in an investigation of 197 patients conducted in 2018. Thereafter, we increased the sample size from 197 to 234 patients and analyzed the findings for all 234 patients to further investigate the association of suicide-related behaviors with levels of trace lithium, EPA, DHA, and AA. Moreover, we used new statistical analyses to avoid multicollinearity.

## 2 Subjects and methods

### 2.1 Study design and subjects

The present study consecutively collected data from 19 patients in a university hospital from October 1, 2017 to September 20, 2021, and 18 patients in a psychiatric medical center of a prefectural hospital from October 1, 2020 to March 31, 2022. The scale of the prefectural hospital and that of the university hospital were comparable because both hospitals had about 600 beds. Moreover, four psychiatrists (MK, IS, MSh, and MSa) had moved from the university hospital to the prefectural hospital. Therefore, the study conditions and methods were very similar to those reported in our previous study (29). As for patient recruitment, the inclusion criteria (age ≥ 20 years and alive at the commencement of the study) and exclusion criteria (a history of schizophrenia, lithium therapy, or omega-3 supplementation) were the same as in our previous study (29).

As used in our previous study, to differentiate deliberate self-harm and suicide attempts, we used the revised nomenclature by Silverman et al. (30) to study suicide and suicidal behaviors, whereby the use of the term suicide attempt relates specifically to a self-inflicted act with the intent to end one’s life and is distinguished from deliberate self-harm. Also, the control group suffered from accidental injury or intoxication as described in our previous study (29). Our previous study (29) had been approved by the ethics committee of Oita University Hospital (#619), and all patients gave informed consent, and this study was approved by the ethics committee of Oita Prefectural Hospital (#2–40), and all patients gave informed consent.

### 2.2 Procedures

At the psychiatric medical center, similar to the method used in a university emergency department (29), routine blood sampling was performed at the first visit. After individual patients recovered, informed consent was obtained and, if they agreed, we used a remnant of the blood to measure serum lithium and levels of plasma EPA, DHA, and AA. Plasma EPA, DHA, and AA levels were measured by a third-party laboratory (Special Reference Laboratories) using gas-chromatography, which could measure EPA, DHA, and AA levels as low as 0.2, 0.3, and 0.2 μg/mL, respectively. Serum lithium levels were measured by one of the author (TM) who was blinded to the data of the patients using mass spectrometry, in which the minimum amount of lithium that could be measured was 0.15 μg/L. The distributions of lithium, EPA, DHA, and AA levels were deviated, and therefore log transformation was employed to use parametric statistical procedures.

### 2.3 Statistical analysis

Patient characteristics, mean EPA, DHA, AA, and lithium levels, and mean log-transformed values were compared between the suicide-attempt group, deliberate self-harm group, and control group in a one-way analysis of variance (ANOVA) with a *post-hoc* test and by the Bonferroni test for continuous variables and χ2 test for categorical variables. Considering the possibility of multicollinearity among EPA, DHA, and AA because of the potential interactions between omega-3 and omega-6 fatty acids, we calculated the Pearson correlation coefficient values. In the present samples, the log-transformed EPA, DHA, and AA levels showed significant associations. In particular, log-transformed DHA levels showed a very high significant association with log-transformed EPA levels (*r* = 0.84, *p* < 0.001) and a significant association with log-transformed AA levels (*r* = 0.38, *p* < 0.001). Moreover, log-transformed AA levels also showed a significant association with log-transformed EPA levels (*r* = 0.29, *p* < 0.001). Conversely, log-transformed lithium levels showed no significant association with log-transformed EPA, DHA, or AA levels. Therefore, we decided to separately perform three multivariate logistic regression analyses that included log-transformed EPA and lithium levels in the first model, log-transformed DHA and lithium levels in the second model, and log-transformed AA and lithium levels in the third model to avoid multicollinearity that could have distorted the results. All models included age and sex as covariates. As such, three multivariate logistic regression analyses were performed to determine whether suicide attempts and deliberate self-harm were predicted by age, sex, lithium levels, and other factors. Data were analyzed with SPSS version 27 for Windows.

## 3 Results

After including data from 37 patients, the total number of the study participants increased from 197 to 234, with the number of patients in the suicide-attempt group increasing from 33 to 39, the number of patients in the deliberate self-harm group increasing from 18 to 29, and the number of patients in the control group increasing from 146 to 166.

With regard to psychiatric diagnoses, the control group (*N* = 166) included participants with depression (*N* = 3), dementia (*N* = 3), others (*N* = 6), none (*N* = 134), and lack of data (*N* = 20). The deliberate self-harm group (*N* = 29) included participants with depression (*N* = 9), borderline personality disorder (*N* = 3), bipolar disorder (*N* = 3), others (*N* = 6), none (*N* = 1), and lack of data (*N* = 7). The suicide attempt group (*N* = 39) included participants with depression (*N* = 13), bipolar disorder (*N* = 5), adjustment disorder (*N* = 2), dementia (*N* = 2), others (*N* = 6), none (*N* = 5), and lack of data (*N* = 6).

As for medications, the participants in the control group were administered antipsychotics (*N* = 0), antidepressants (*N* = 4), mood stabilizers (*N* = 2), and benzodiazepines (*N* = 7) [none (*N* = 128) and lack of data (*N* = 25)]. The participants in the deliberate self-harm group were administered antipsychotics (*N* = 6), antidepressants (*N* = 11), mood stabilizers (*N* = 5), and benzodiazepines (*N* = 14) [none (*N* = 2), and lack of data (*N* = 8)], whereas the participants in the suicide attempt group were administered antipsychotics (*N* = 4), antidepressants (*N* = 7), mood stabilizers (*N* = 1), and benzodiazepines (*N* = 18) [none (*N* = 12) and lack of data (*N* = 6)]. Several patients had a combination of drugs.

**FIGURE 1:**
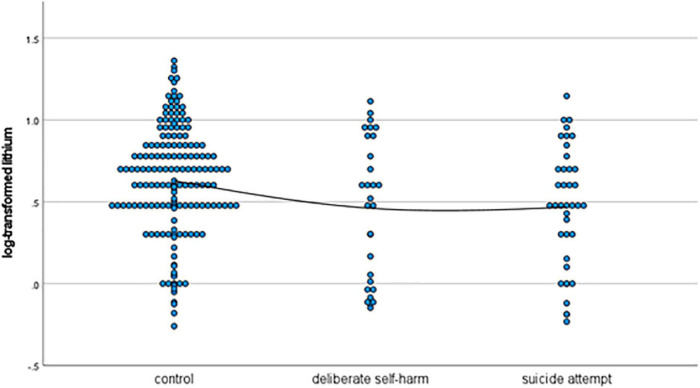
Scattergram of log-transformed lithium levels in the control, deliberate self-harm, and suicide-attempt groups.

**FIGURE 2:**
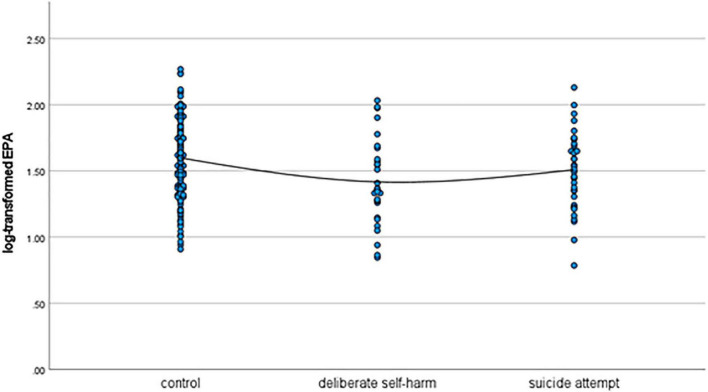
Scattergram of log-transformed EPA levels in the control, deliberate self-harm, and suicide-attempt groups.

**FIGURE 3:**
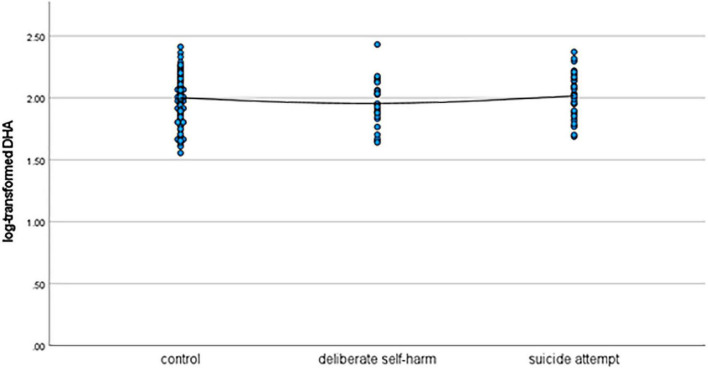
Scattergram of log-transformed DHA levels in the control, deliberate self-harm, and suicide-attempt groups.

**FIGURE 4:**
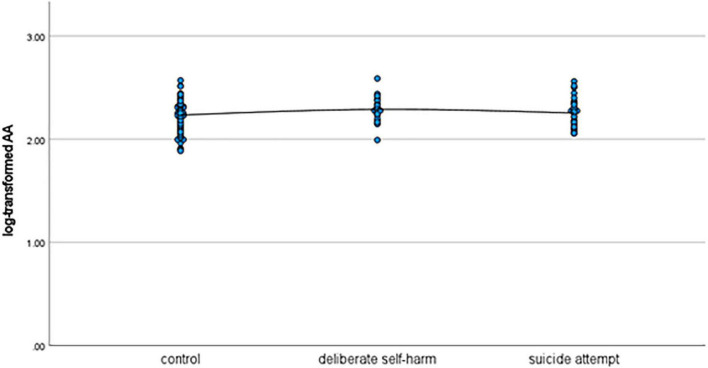
Scattergram of log-transformed AA levels in the control, deliberate self-harm, and suicide-attempt groups.

As shown in Table 1, patients in the deliberate self-harm group were significantly younger than those in the control group. Moreover, the deliberate self-harm group had significantly more female patients than the control group. The mean log-transformed lithium level was significantly lower in the suicide-attempt group than in the control group. The mean log-transformed EPA level was significantly lower in the deliberate self-harm group than in the control group. Figures 1–4 show the scattergrams of log-transformed lithium, EPA, DHA, and AA levels in the control group, deliberate self-harm group, and suicide-attempt group, respectively.

**TABLE 1:** The characteristics of patients and lithium, EPA, DHA, AA levels and their log-transformed values in the suicide-attempt group, deliberate self-harm group, and control group.

	Suicide-attempt group	Deliberate self-harm group	Control group	*P*	*Post hoc*
Number	39	29	166		
Age	49.6 (18.6)	43.6 (14.9)	54.3 (20.5)	0.018	Deliberate self-harm < control
Sex (F:M)	16:23	18:11	55:111	0.011	
Lithium (μg/L)	4.04 (3.16)	4.37 (3.68)	5.62 (4.26)	0.045	
Log-transformed lithium	0.47 (0.37)	0.46 (0.43)	0.63 (0.34)	0.007	Suicide attempt < control
EPA (μg/mL)	39.4 (26.2)	34.4 (27.8)	48.1 (31.1)	0.036	
Log-transformed EPA	1.51 (0.29)	1.42 (0.32)	1.60 (0.28)	0.004	Deliberate self-harm < control
DHA (μg/mL)	112.5 (45.4)	99.5 (47.2)	108.1 (41.5)	0.46	
Log-transformed DHA	2.02 (0.18)	1.96 (0.19)	2.00 (0.17)	0.35	
AA (μg/mL)	187.8 (60.5)	200.9 (53.7)	178.3 (50.5)	0.086	
Log-transformed AA	2.25 (0.13)	2.29 (0.11)	2.23 (0.17)	0.080	

As shown in Table 2, the first multivariate logistic regression analysis with adjustment for age, sex, and log-transformed lithium and EPA levels revealed that the negative association with log-transformed lithium levels was significantly greater in patients with suicide attempts than in control patients, and that the negative association with EPA levels and the positive association with female sex were significantly greater in patients with deliberate self-harm than in control patients.

**TABLE 2:** Multivariate logistic regression analyses of lithium and EPA in the suicide-attempt group and deliberate self-harm group with the control group as a reference.

	Odds ratio (95% CI)	*P*
**Suicide-attempt group**		
Age	1.00 (0.98–1.02)	0.63
Sex (female = 0, male = 1)	1.40 (0.67–2.91)	0.37
Log-transformed lithium	0.32 (0.12–0.86)	0.023
Log-transformed EPA	0.46 (0.11–1.89)	0.28
**Deliberate self-harm group**		
Age	0.99 (0.96–1.01)	0.28
Sex (female = 0, male = 1)	3.34 (1.42–7.84)	0.006
Log-transformed lithium	0.36 (0.11–1.13)	0.08
Log-transformed EPA	0.18 (0.032–0.98)	0.047

Reference: control group.

The goodness of fit of the model was significant (χ^2^ = 28.4, *p* < 0.001).

As shown in Table 3, the second multivariate logistic regression analysis with adjustment for age, sex, and log-transformed lithium and DHA levels revealed that the negative association with log-transformed lithium levels was significantly larger in patients with suicide attempts and in patients with deliberate self-harm than in control patients, and that the positive association with female gender was significantly larger in patients with deliberate self-harm than in control patients. DHA levels showed no significant association with suicide attempts or deliberate self-harm.

**TABLE 3:** Multivariate logistic regression analyses of lithium and DHA in the suicide-attempt group and deliberate self-harm group with the control group as a reference.

	Odds ratio (95% CI)	*P*
**Suicide-attempt group**		
Age	0.98 (0.96–1.00)	0.097
Sex (female = 0, male = 1)	1.30 (0.62–2.71)	0.48
Log-transformed lithium	0.29 (0.11–0.77)	0.013
Log-transformed DHA	5.57 (0.51–61.4)	0.16
**Deliberate self-harm group**		
Age	0.98 (0.95–1.00)	0.051
Sex (female = 0, male = 1)	3.12 (1.34–7.26)	0.008
Log-transformed lithium	0.31 (0.10–0.96)	0.042
Log-transformed DHA	0.91 (0.05–15.2)	0.95

Reference: control group.

The goodness of fit of the model was significant (χ^2^ = 26.4, *p* < 0.001).

As shown in Table 4, the third multivariate logistic regression analysis with adjustment for age, sex, and log-transformed lithium and AA levels revealed that the negative association with log-transformed lithium levels was significantly greater in patients with suicide attempts and in patients with deliberate self-harm than in control patients, and that the positive association with AA levels and female sex and the negative association with age were significantly larger in patients with deliberate self-harm than in control patients.

**TABLE 4:** Multivariate logistic regression analyses of lithium and AA in the suicide-attempt group and deliberate self-harm group with the control group as a reference.

	Odds ratio (95% CI)	*P*
**Suicide-attempt group**		
Age	0.99 (0.97–1.01)	0.29
Sex (female = 0, male = 1)	1.37 (0.66–2.85)	0.40
Log-transformed lithium	0.30 (0.11–0.81)	0.017
Log-transformed AA	3.09 (0.17–54.9)	0.44
**Deliberate self-harm group**		
Age	0.98 (0.95–1.00)	0.026
Sex (female = 0, male = 1)	3.33 (1.42–7.81)	0.006
Log-transformed lithium	0.32 (0.10–0.98)	0.045
Log-transformed AA	45.3 (1.22–1681.2)	0.039

Reference: control group.

The goodness of fit of the model was significant (χ^2^ = 28.9, *p* < 0.001).

## 4 Discussion

The main findings of the present study were as follows: higher log-transformed lithium levels were significantly associated with fewer suicide attempts and lower deliberate self-harm; higher log-transformed EPA levels were significantly associated with lower deliberate self-harm; higher log-transformed AA levels were significantly associated with higher deliberate self-harm; and the log-transformed DHA level was not associated with suicide attempts or deliberate self-harm. These findings suggest that naturally absorbed lithium may be protective against suicide and deliberate self-harm; naturally absorbed EPA may be protective against deliberate self-harm; and naturally absorbed AA may be a risk factor for deliberate self-harm.

Our previous study (29) suggested that both EPA and lithium may be protective factors against suicide attempts, whereas DHA may be risk factor for suicide attempt, and that AA may be a risk factor for deliberate self-harm. The effects of lithium against suicide attempts were consistently supported by both the present and previous study and seem to be robust. In addition, lithium may also be effective against deliberate self-injury, which was significantly confirmed in the second model that included lithium and DHA levels as independent factors (Table 3) and in the third model that included lithium and AA levels as independent factors (Table 4) and showed a significant tendency (*p* = 0.08) in the first model that included lithium and EPA levels as independent factors (Table 2). Surprisingly, lithium may be effective for both suicide attempt and deliberate self-injury. Moreover, AA as a risk factor for deliberate self-harm was confirmed and reconfirmed by both studies and may be robust. On other hand, the effects of EPA against suicide attempts (29) may have been a false-positive finding possibly attributed to multicollinearity with DHA and AA. In the present study, the effects of EPA may not be against suicide attempt but against deliberate self-harm, which was not significant in the previous study, but excluding DHA and AA from the analysis and increasing the number of participants may have yielded such findings. Moreover, DHA as a risk factor for suicide attempt (29) was not reconfirmed in the present study, which may have been one of the false-positive findings probably caused by multicollinearity with EPA and AA. Similar to the case of EPA, exclusion of EPA and AA from the analysis and increasing the number of participants may have yielded such findings.

The limitations of the study are as follows: First, the participants were cases of suicide attempts and not those who had committed suicide. Therefore, the present findings cannot be extrapolated to suicide completers. Second, the sample size of this study was only slightly larger than the previous study. Nonetheless, the present results were clearly different from the previous ones due to the different statistical analyses that avoided multicollinearity. Third, we differentiated suicide attempt from deliberate self-harm from the point of view of the intent to end one’s life. That is, if the intent to end one’s life was present during the event, we considered it as a suicide-attempt, and if not, we considered it as deliberate self-harm. However, there might be a situation that the patients could not recall the intent during the event, although there had been actual intent. Fourth, although lithium and one of the EPA, DHA, and AA were paired in the multivariate logistic regression analyses to avoid multicollinearity, we could not adjust EPA, DHA, AA, and lithium as a whole. Fifth, the reasons and mechanisms accounting for the differences of the effects of EPA, DHA, AA, and lithium are unknown. Sixth, the effects of medications on polyunsaturated fatty acid concentrations, if any, were not adjusted. Finally, the present findings were not in agreement with the findings of a large randomized clinical trial (7). There have been conflicting findings on the effects of omega-3 on mental state. Some reports supported the effects (31, 32), whereas others dismiss the effects (7, 33). Unfortunately, the reason for this discrepancy is unknown, but the present findings support the effects of omega-3. Especially, the significant effects of EPA and non-significant effects of DHA are consistent with the findings of Liao et al.’s (31) meta-analysis. Further studies are required to resolve the discrepancy.

In conclusion, the present findings suggest that naturally absorbed lithium may be protective against suicide and deliberate self-harm while naturally absorbed EPA may be protective against deliberate self-harm, and that naturally absorbed AA may be a risk factor for deliberate self-harm.

## Data availability statement

The raw data supporting the conclusions of this article will be made available by the authors, upon reasonable request.

## Ethics statement

Our previous study had been approved by the Ethics Committee of Oita University Hospital (#619), and all patients gave informed consent, and this study was approved by the Ethics Committee of Oita Prefectural Hospital (#2–40), and all patients gave informed consent.

## Author contributions

TI, MK, and TT participated in study design. MK, IS, MSh, and MSa collected data. TM measured serum lithium levels. All authors constructively criticized the manuscript, were responsible for writing the manuscript, and read and approved the final version of the manuscript for publication.
